# Circular RNAs as biomarkers for lung cancer

**DOI:** 10.1016/j.ncrna.2022.11.002

**Published:** 2022-11-07

**Authors:** Albert Sufianov, Sema Begliarzade, Aferin Beilerli, Yanchao Liang, Tatiana Ilyasova, Ozal Beylerli

**Affiliations:** a Educational and Scientific Institute of Neurosurgery, Рeoples’ Friendship University of Russia (RUDN University), Moscow, Russia; b Department of Neurosurgery, Sechenov First Moscow State Medical University (Sechenov University), Moscow, Russia; c Republican Clinical Perinatal Center, Ufa, Republic of Bashkortostan, 450106, Russia; d Department of Obstetrics and Gynecology, Tyumen State Medical University, 54 Odesskaya Street, 625023, Tyumen, Russia; e Department of Neurosurgery, The First Affiliated Hospital of Harbin Medical University, Harbin, 150001, China; f Department of Internal Diseases, Bashkir State Medical University, Ufa, Republic of Bashkortostan, 450008, Russia

**Keywords:** Noncoding RNAs, Circular RNAs, Lung cancer, Biology function, Biomarker

## Abstract

Lung cancer is the leading cause of death and morbidity from malignant neoplasms worldwide, and its poor prognosis places a heavy burden on patients. A large percentage of lung cancer cases are associated with smoking. A significant number of non-smokers also develop the disease, suggesting an epigenetic and genetic mechanism for the development of lung cancer. The current situation with the diagnosis and treatment of lung cancer remains grim, and effective therapeutic targets and molecular markers are urgently needed. Circular RNAs (circRNAs) are covalently closed non-coding RNAs that have received much attention due to their biological properties such as conservatism, stability, and tissue specificity. Many studies have shown that circRNAs are involved in the regulation of lung cancer through various mechanisms, such as microRNA adsorption, and play an important role in the early diagnosis, treatment, and prognosis of lung cancer. In recent years, it has become increasingly clear that circRNAs are involved in the proliferation, migration, and invasion of lung cancer cells. Differentially expressed circRNAs can be used as non-invasive diagnostic and prognostic markers of lung cancer. This article summarizes the current advances of circRNAs in the diagnosis, treatment and prognosis of lung cancer.

## 1 Introduction

Lung cancer is the most common malignant tumor disease in the world, posing a serious threat to human life and health. According to statistics, in 2018, there were about 2.1 million new cases of lung cancer and 1.8 million deaths from lung cancer worldwide, with the morbidity and mortality ranking first among all cancer types [1]. According to histological types, lung cancer can be divided into small cell lung cancer and non-small cell lung cancer, among which small cell lung cancer and non-small cell lung cancer account for about 15% of the total lung cancers, respectively and 85% [2]. Although clinical diagnosis and treatment methods have improved, the 5-year survival rate of lung cancer is still not optimistic due to untimely diagnosis, limited beneficiary population, and drug resistance of patients. In addition, the lack of relatively specific tumor markers adds challenges to the diagnosis, treatment and prognosis of lung cancer. Therefore, it is necessary to deeply study the molecular mechanism of lung cancer to explore potential biomarkers and therapeutic targets for lung cancer.

Circular RNA (circRNA) is a special kind of endogenous non-coding RNA. As early as the 1970s, circRNA was found to exist in RNA viruses [3,4]. However, due to the limitations of the technology at the time, circRNAs were considered to be by-products of the splicing process, so they did not receive widespread attention [5]. In recent years, with the development of high-throughput sequencing technology and bioinformatics, circRNAs have been discovered in large numbers and gradually become a research hotspot in the field of RNA. At present, many studies have confirmed that circRNA can participate in the regulation of the occurrence and development of lung cancer, and is expected to provide new ideas for the diagnosis, treatment and prognosis of lung cancer [[6], [7], [8], [9]].

## 2 Biological functions of circular RNAs

**Fig. 1:**
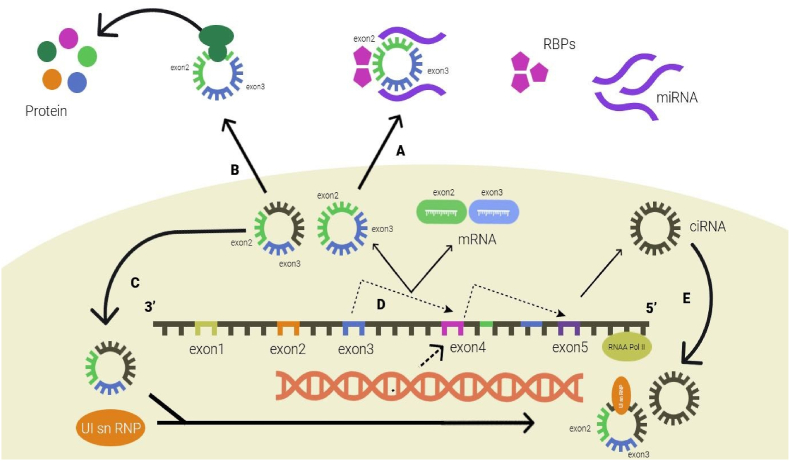
Biological functions of circular RNAs. (A) circRNAs act as miRNA sponges. (B) circRNAs bind to proteins, such as RBP and MBL. (C) circRNAs act as translation templates. (D) circRNAs regulate transcription. (E) circRNAs regulate protein expression.

CircRNAs are covalently closed noncoding RNA molecules that are ubiquitous in eukaryotic transcriptomes. CircRNAs are usually divided into exonic circRNAs (ecRNAs), intronic circRNAs (ciRNAs), and exon-intron circRNAs (EIciRNAs) according to their sources [10]. Among them, exonic circRNAs are the most common. Unlike linear RNA, circRNA does not have a cap structure at the 5′ end and a polyadenylation tail at the 3′ end, which can resist the degradation of exonuclease RNase R, so circRNA is more stable and has a longer half-life than linear RNA [11]. Research also found that circRNAs show good species conservation [12]. In addition, the expression of circRNAs is tissue specific and developmental stage specific, suggesting that circRNAs may be involved in the regulation of various pathophysiological processes in the body [13]. The functional studies of circRNAs mostly focus on the following aspects: 1) Adsorb miRNAs as molecular sponges. Competing endogenous RNA (ceRNA) mechanism points out that RNAs with the same miRNA response elements (MREs) can competitively bind miRNAs, thereby regulating each other's expression (Fig. 1) [14].

At present, most circRNAs studies focus on the mechanism of miRNA molecular sponge; 2) regulate the transcription of parental genes by binding to RNA polymerase II [15]; 3) interact with RNA-binding proteins to play biological roles (Table 1) [12]; 4) translate proteins. For example, Yang et al. found that Circ-FBXW7 encodes a protein that inhibits the occurrence of gliomas [26].

**Table 1:** Functions of circRNAs.

Function	Example	Ref.
miRNA sponge	circ-HIPK3 circ-PRKCI	[16,17]
Histone methylation	Circ-ANRIL	[18]
Protein sponge	circ-Foxo3	[19]
RNA maturation	circ-ANRIL	[20]
RNAP II elongation	circ-EIF3J circ-PAIP2	[21]
Translation regulator	circ-PABPN1	[22]
Alternative splicing	circ-Mbl	[23]
Protein translation (including m6A-driven)	circ-ZNF609	[24,25]

## 3 circRNAs and the diagnosis of lung cancer

Early and accurate diagnosis is critical to the treatment of lung cancer. Although a variety of diagnostic methods have been used in clinical practice, the current methods still have room for improvement due to reasons such as cost, accuracy, and patient acceptance. Therefore, it is still necessary to explore the diagnostic markers of lung cancer. CircRNAs have the advantages of conservation, stability, and specificity, so they have the potential to become emerging markers of lung cancer [27]. A meta-analysis of the Chinese lung cancer population pooled 8 studies on the diagnostic efficacy of circRNAs in lung cancer tissue and blood. The area under curve (AUC) of characteristic curve (ROC) was 0.78, suggesting that circRNAs have diagnostic potential in the Chinese lung cancer population [28].

### 3.1 The diagnostic value of blood circRNAs

Compared with traditional biopsy, liquid biopsy has the advantages of simple operation, less invasiveness, and low cost, so the research prospect is broad. At present, some literatures have preliminarily confirmed that plasma circRNAs have good diagnostic ability, such as circRNA-002178, circMAN1A2 and so on [29,30]. Chen et al. used high-throughput sequencing technology to identify differentially expressed circRNAs in plasma exosomes from lung adenocarcinoma (LUAD) patients [31]. Compared with the control group, the expression of 105 circRNAs was increased, and the expression of 78 circRNAs was decreased. Further research found that the expressions of hsa_circ_0001492 and hsa_circ_0001346 were significantly up-regulated in the early stage of LUAD, but were almost undetectable in the plasma of the control group, suggesting that hsa_circ_0001492 and hsa_circ_0001346 may be candidate markers for early LUAD diagnosis. Liu et al. detected and analyzed the differential expression of hsa_circ_0005962 and hsa_circ_0086414 in the plasma of LUAD patients [32]. The combined diagnosis AUC of the two reached 0.81, suggesting that dual circRNAs may be used as non-invasive biomarkers for the diagnosis of LUAD. In addition, blood circRNA may be related to tumor progression, and the expression of hsa_circ_0005962 in LUAD patients was significantly decreased after surgery compared with preoperative ones. The expression level of hsa_circ_0086414 was correlated with epidermal growth factor receptor (EGFR) mutation. Compared with wild-type patients, hsa_circ_0086414 was highly expressed in EFGR mutant patients. This study demonstrates the multi-faceted application value of blood circRNAs. Of course, in order to realize the clinical translation of blood circRNA lung cancer diagnosis, a larger sample size and more in-depth mechanism exploration are still needed.

### 3.2 Diagnostic value of circRNAs in lung cancer tissues

**Fig. 2:**
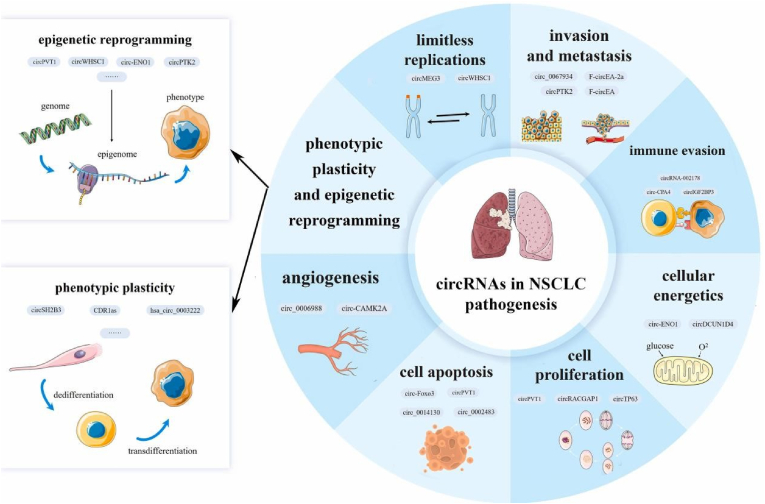
The role of circRNAs in the pathogenesis of non-small cell lung cancer.

Wang et al. found that in distinguishing non-small cell lung cancer from normal tissues, the AUCs of hsa_circ_0077837 and hsa_circ_0001821 were 0.921 and 0.863, respectively, showing the diagnostic value of these two circRNAs for lung cancer [33]. Liu et al. confirmed that the expression of hsa_circ_11780 was significantly decreased in non-small cell lung cancer tissues and cell lines, and patients with low expression of hsa_circ_11780 had a greater risk of developing larger tumors (>3 cm), distant metastasis and poor survival prognosis [34]. Zhao et al. analyzed 61 pairs of paired lung cancer and paracancerous tissues and found that hsa_circ_0037515 and hsa_circ_0037516 were lowly expressed in non-small cell lung cancer, and their AUCs were 0.81 and 0.82, respectively, which also showed good diagnostic ability (Fig. 2) [35]. The combined AUC of hsa_circ_0037515 and hsa_circ_0037516 increased to 0.90, indicating the importance of circRNA joint diagnosis in lung cancer tissue.

## 4 circRNAs and the treatment of lung cancer

Previous studies have found that circRNA can act as a regulatory molecule to promote or inhibit the occurrence and development of lung cancer, so regulating the expression level of circRNA is of great significance to the malignant biological behavior of lung cancer. At present, many studies have explored the mechanism of malignant biological behavior of lung cancer based on the ceRNA mechanism of circRNAs (Table 2).

**Table 2:** Summary of circRNA acting on malignant biological behaviors of lung cancer through ceRNA mechanism.

circRNA	Dysregulation	Cell lines	Function	Sponge target	Ref.
circ_11780	Down	A549, H226	Proliferation (−), migration (−), invasion (−)	miR-544a	[34]
circGFRA1	Up	A549, H838	Proliferation (+)	miR-188–3p	[36]
circ_0012673	Up	A549, H23	Proliferation (+), apoptosis (−), migration (+), EMT (+)	miR-320a	[38]
circ-0000211	Up	A549, H1299, H1650	Migration (+), invasion (+)	miR-622	[39]
circ-ABCB10	Up	A549, H292	Proliferation (+), migration (+)	miR-556–3p	[40]
circ_0000326	Up	A549, H1299	Proliferation (+), apoptosis (−), migration (+)	miR-338–3p	[41]
circ_0014130	Up	PC-9, A549	Proliferation (+), apoptosis (−), invasion (+)	miR-136–5p	[42]
circ-SOX4	Up	A549, SPC-A1	Proliferation (+), migration (+), invasion (+)	miR-1270	[43]
circCCDC66	Up	A549, H1299	Proliferation (+), apoptosis (−), migration (+), invasion (+)	miR-33a-5p	[44]
circCDR1as	Up	A549, Calu-3	Proliferation (+), apoptosis (−), migration (+), invasion (+)	miR-219a-5p	[45]
circ_0058124	Up	A549, H1975	Proliferation (+), apoptosis (−), migration (+), invasion (+)	miR-1297	[46]
circ-MTO1	Down	A549, SPC-A1	Proliferation (−)	miR-17	[47]
cMras	Down	A549, H1299	Proliferation (−), migration (−)	miR-567	[48]
circ-IGF1R	Down	PC9, A549	Migration (−), invasion (−)	miR-1270	[49]
circCRIM1	Down	A549, H1299, SPC-A1	Migration (−), invasion (−)	miR-93, miR-182	[50]
circ_0007059	Down	A549, H1975	Proliferation (−), EMT (−)	miR-378	[51]
circ_0006427	Down	SPC-A1, Calu-3	Proliferation (−), migration (−), invasion (−)	miR-6783–3p	[52]
circPTPRA	Down	H23, H1755, H522	Migration (−), invasion (−), EMT (−)	miR-96–5p	[53]
circSMARCA5	Down	A549	Proliferation (−), migration (−), invasion (−)	miR-19b-3p	[54]
circ_0002483	Down	A549, H1299	Proliferation (−), migration (−), invasion (−)	miR-182–5p	[55]
circ_0078767	Down	A549, H23	Proliferation (−), apoptosis (+), invasion (−)	miR-330–3p	[56]

For example, Yao et al. found that circGFRA1 was up-regulated in lung cancer cells and promoted the malignant proliferation of lung cancer through the circGFRA1/miR-188–3p/PI3K/AKT pathway [36]. As a serine-threonine protein kinase, LIMK1 participates in epithelial-mesenchymal transition (EMT) by affecting the actin cytoskeleton and regulates tumor progression [37]. Qin et al. found that circ_0012673 was highly expressed in lung cancer tissues and cell lines [38]. The adsorption of miR-320a by circ_0012673 sponge resulted in increased expression of the downstream target protein LIMK1, thereby inhibiting lung cancer cell apoptosis and promoting its proliferation, migration and EMT process.

### 4.1 circRNAs and lung cancer immunotherapy

Tumor cells are able to express a variety of mechanisms to evade the immune system and create conditions for their own growth. Programmed death protein 1 (PD-1) is a transmembrane protein, which has been found to be expressed on the surface of almost all types of tumor cells, and participates in tumor immune escape by interacting with PD-L1 mechanism [57]. In recent years, immune checkpoint inhibitors (ICIs) targeting PD-1/PD-L1 have provided a powerful weapon for lung cancer treatment Wang et al. found that circRNA-002178 was abnormally highly expressed in lung adenocarcinoma tissues, and promoted the expression of PD-L1 in lung cancer cells by adsorbing miR-34 [29]. At the same time, lung cancer cells can secrete exosomal circRNA-002178 and deliver it to T cells, which promotes the expression of PD-1 in T cells by inhibiting miR-28–5p. Literature confirmed that CXCR4 is involved in the process of cytotoxic T lymphocyte depletion and induction of anti-PD-1 drug resistance [58]. Zhang et al. found that the circFGFR1/miR-381–3p/CXCR4 pathway plays an immunosuppressive effect by promoting the resistance of lung cancer cells to anti-PD-1 drugs [59]. It is suggested that circRNAs can participate in tumor immune escape mechanism, and the combined use of related pathway inhibitors is expected to improve clinical efficacy and provide new ideas for tumor immunotherapy.

### 4.2 circRNAs and drug resistance in lung cancer

With the continuous advent of anti-tumor drugs, it has brought more hope to lung cancer patients, but the problem of drug resistance is still a major problem that plagues clinical treatment. Therefore, it is urgent to further explore the drug resistance mechanism of lung cancer in order to find efficient biomarkers or therapeutic targets. Studies have found that some circRNAs can participate in the drug resistance process of lung cancer (Table 3).

**Table 3:** Summary of the effects of circRNA on tumor drug sensitivity.

CircRNA	Cell lines	Drugs	Sensitivity	Ref.
circAKT3	A549, H1299	Cisplatin	Down-regulated	[6]
circ-ABCB10	A549, H292	Cisplatin	Down-regulated	[40]
circ_0002483	A549, H1299	Paclitaxel	Up-regulated	[55]
circZFR	A549, H522	Cisplatin	Down-regulated	[62]
circ_0076305	A549, H1650	Cisplatin	Down-regulated	[63]
circ_0004015	A549, HCC827	Gefitinib	Down-regulated	[64]
circ_0003998	A549, H1299	Docetaxel	Down-regulated	[65]
circ_0001946	A549	Cisplatin	Up-regulated	[66]
circESRP1	H69, H446	Cisplatin, etoposide	Up-regulated	[67]
circ-SMARCA5	H1299, H1437	Cisplatin, gemcitabine	Up-regulated	[68]

Hong et al. found that circCPA4 acts as a molecular sponge of let-7, and its down-regulation can affect programmed death-ligand 1 (PD-L1) to reduce its expression, thereby inhibiting the growth and development of non-small cell lung cancer cells migration and EMT process [60]. In addition, non-small cell lung cancer-derived PD-L1-containing exosomes can promote their stem cell properties and enhance the tolerance of non-small cell lung cancer cells to cisplatin. Li et al. reported that circ_0002483 could reduce the expression level of miR-182–5p, relieve its inhibition of target molecules GRB2, FOXO1, and FOXO3, thereby enhancing the sensitivity of non-small cell lung cancer to paclitaxel [55]. CircRNA_103762 is highly expressed in lung cancer and induces multidrug resistance in lung cancer by inhibiting the target protein CHOP [61].

## 5 circRNAs and prognosis of lung cancer

Prognostic monitoring of patients with lung cancer is a key link in evaluating the effect of clinical diagnosis and treatment, and is of great significance for adjusting drug regimens and improving patient survival time. Studies have confirmed that a variety of circRNAs can be used as independent prognostic indicators of lung cancer patients and are closely related to the survival of lung cancer patients, such as circSMARCA5, circ_11780, circCRIM1 [30,50,68]. Liu et al. performed RT-qPCR detection on tumor tissues of 93 non-small cell lung cancer patients and found that hsa_circ_11780 was abnormally low expressed, and patients with low expression of hsa_circ_11780 tended to have larger tumors with distant metastasis and more severe tumor according to tumor-lymph node- Metastasis (TNM) staging [34]. Survival analysis by Kaplan-Meier method showed that non-small cell lung cancer patients with low expression of hsa_circ_11780 had shorter overall survival (OS). circHIPK3 is derived from exon 2 of the oncogene HIPK3 in the chromosome 11p13 region. Chen et al. found that knockdown of circHIPK3 could inhibit the proliferation, migration, and invasion of non-small cell lung cancer cell lines A549, H838, and H1299, and induce the occurrence of autophagy, while circHIPK3 and linHIPK3 antagonized the regulation of autophagy [9]. CircHIPK3:linHIPK3 (C:L) ratio can reflect the autophagy level of tumor cells. For patients with advanced non-small cell lung cancer, high C:L ratio (>0.49) is an effective indicator of low survival rate. These results suggest that the autophagy regulator circHIPK3 has potential clinical application value as a prognostic factor. EGFR-tyrosine kinase inhibitors (EGFR-TKIs) are an important treatment option for non-small cell lung cancer patients with sensitive EGFR mutations. Liu et al. detected 1377 differentially expressed circRNAs by sequencing the plasma circRNAs of non-small cell lung cancer patients in the effective and ineffective groups after using EGFR-TKI gefitinib [69]. RT-qPCR detection confirmed that hsa_circ_0109320 and hsa_circ_0134501 were highly expressed in the gefitinib effective group. Further research found that the high expression of hsa_circ_0109320 was associated with better progression-free survival (PFS) in patients, suggesting that hsa_circ_0109320 may be a biomarker reflecting the efficacy of gefitinib. Fu et al. found that the expression of hsa_circRNA_012515 was significantly increased in non-small cell lung cancer tissues and cells, especially in gefitinib-resistant cell lines [70]. In addition, the up-regulation of hsa_circRNA_012515 was closely related to lymph node metastasis, tumor stage and prognosis of patients. Non-small cell lung cancer patients with high expression of hsa_circRNA_012515 had shorter OS and PFS. We also found that hsa_circRNA_012515 was expressed at higher levels in stage III/IV patients compared with stage I/II non-small cell lung cancer patients. Thus, hsa_circRNA_012515 has good clinical correlation and may be a biomarker for predicting poor prognosis of non-small cell lung cancer patients.

## 6 Conclusions

With the deepening of research, the connection between circRNA and lung cancer is becoming increasingly prominent. On the one hand, circRNAs act as tumor-promoting or tumor-suppressing factors to regulate the biological behaviors of lung cancer, such as proliferation, metastasis, apoptosis, and autophagy, regulate the sensitivity of chemotherapy or targeted drugs and the efficacy of immunotherapy, and provide a preliminary theoretical basis for adjuvant clinical treatment. On the other hand, the differential expression of circRNAs in tissue or blood shows a certain correlation in the early diagnosis and prognosis evaluation of lung cancer, and is expected to become a potential biomarker of lung cancer. However, the current circRNA research is still in the early stage, most researchers focus on the exploration of the adsorption function of miRNA sponges, and many mechanisms have not yet been elucidated [[71], [72], [73], [74], [75]]. Its clinical relevance research is also limited to a small number of samples, and its translational value remains to be questioned. It is believed that there will be more breakthroughs in the field of circRNA in the future, providing more ideas for the diagnosis and treatment of lung cancer.

## Funding

This work was supported by the Bashkir State Medical University Strategic Academic Leadership Program (PRIORITY-2030).

## Author contributions

Albert Sufianov: Conceptualization, Writing – original draft, Project administration. Sema Begliarzade: Writing – review & editing, Investigation, Project administration. Aferin Beilerli: Formal analysis, Methodology, and original draft. Yanchao Liang: Resources, Data curation. Tatiana Ilyasova: Validation, Visualization. Ozal Beylerli: Supervision, Funding acquisition. All authors have read and agreed to the published version of the manuscript.

## Declaration of competing interest

The authors declare that the research was conducted in the absence of any commercial or financial relationships that could be construed as a potential conflict of interest.
